# Correspondence

**Published:** 1873-04

**Authors:** Wm. R. Whitehead

**Affiliations:** Late of New York; Denver, Colorado


### CORRESPONDENCE.

DENVER, COLORADO, February 21, 1873.

*Editors Southern Medical Record:*

To this remote part of the West the *Georgia Medical Companion* followed me, and on the reception of the December number, I greeted it with the warmth of an old friend; but I was painfully reminded of an unfulfilled promise to contribute an occasional article to this journal.

From motives connected with the health of my family, I have taken up my residence temporarily, perhaps permanently, and have engaged in the practice of medicine at Denver, Colorado. The climate of this Territory is delightful, generally throughout the year, and is a desirable resort for invalids with incipient phthisis, with asthma especially, and some other chronic affections. The changes here, however, are very great and very sudden; nevertheless, there is less inconvenience from such changes than in any part of the United States. The following table, made by Mr. Henry Fenton, of the United States Signal Service, will afford a correct idea of the thermometer, and other changes at Denver, during the winter and spring months:

### TABLE.

| MONTH. | Mean Barometer. | Mean Thermometer. | Amt. Rain or Melted Snow. Inches. | Prevailing Direction of Wind. | Highest Daily Mean Thermometer. | Lowest Daily Mean Thermometer. | REMARKS. |
|---|---|---|---|---|---|---|---|
| 1871. | | | | | | | |
| December, . . . . | 29.788 | 29°.12 | . . . | South. | 46°.00 | 4°.67 | In January and December snow drifted a good deal, and could not be measured accurately. |
| 1872. | | | | | | Minus. | |
| January, . . . . | 30.029 | 23°.82 | . . . | South. | 45°.33 | 11°.13 | |
| February, . . . . | 29.891 | 33°.74 | 0.22 | South. | 47°.83 | 10°.00 | |
| March, . . . . . | 29.959 | 36°.32 | 1.72 | South. | 54°.00 | 19°.50 | |
| April, . . . . . . | 29.889 | 46°.16 | 2.09 | North. | 61°.75 | 29°.50 | |
| May, . . . . . . | 30.009 | 57°.77 | 3.74 | North. | 75°.00 | 40°.25 | |
| June, . . . . . . | 30.044 | 67°.28 | 2.07 | South. | 67°.25 | 51°.50 | |

I do not wish to write an article on the climatology of Colorado, for the very obvious reason that I have not yet resided in it long enough to speak from personal experience of many points of interest connected with the climate. However, this is a subject which has engaged my attention, and upon which I feel inclined to bestow considerable time and thought.

The town, or rather the city, of Denver is delightfully situated in plain view of the snow-capped Rocky Mountains, five thousand feet above the sea. The extreme clearness of the atmosphere makes the snowy range, some seventy or eighty miles distant, appear quite close. The view of these majestic snow-clad mountains, reflecting the bright sunlight, is grand in the extreme, and the sunset views obtained at Denver during the month of October surpass descriptions of the most roseate and poetic kind. Denver is well-built, and indeed contains many beautiful private residences and some fine-looking business structures. The population of this "City of the Plains," as it is called, is about fifteen thousand inhabitants. Denver is an active, rapidly-increasing little city, and presents more attractions in a public and social way than many cities ten times its size. A fine volunteer military organization, called the Governor's Guards, made a display on the streets this afternoon which would deprive the Seventh New York Regiment of their laurels, if submitted to competent judges for a decision with regard to the relative merits of the two organizations. The Guards give a grand military ball to-night in their new armory, a large and elegant brick and stone building. The people of Denver are exceedingly intelligent, and there is a certain air of culture and refinement about the better classes that will very much astonish travelers from the States, who expect to see a population composed mostly of Ute Indians.

The legal and medical professions of Denver are largely represented, and, so far as I can form an opinion, reflect in a most creditable manner the general intelligence which so peculiarly distinguishes this territorial city.

I find that I am writing what might be taken for an *ad captandum* letter, and not furnishing your readers with the article which I propose to send you. If, however, you will accept this informal manner of communicating with them, I will select from my clinic book of the Northwestern Dispensary of New York two or three unusual and important cases, which I believe will prove to be interesting:

*PHAGEDENIC ULCERATION OF THE RECTUM.*

Mrs. S., aged twenty-nine, one child, three miscarriages, came to the Northwestern Dispensary to my clinic February 2, 1871, and the following short notes were recorded in my case-book by Dr. F. M. Deems, my clinical assistant at that time: Mrs. S. has a fœcal and purulent discharge from the anus. By the rectal touch there is not found to be any stricture of the rectum. A speculum examination of the rectum with Sims' speculum revealed deep ulcerative patches of the mucous membrane covering the sphincter ani muscle. The ulcerations were extensive, and encroached on the mucous membrane to a distance considerably above the sphincter. There was an irregular and ulcerated border which corresponded inferiorly to the upper fibres of this muscle. There were soft chancres on and around the vulva. There was a mucous patch three-fourths of an inch in diameter on the right gluteal fold. The ulcerations of the rectum were lightly touched with a solution of chloride of zinc, of one drachm to the ounce of water.

You are aware of the fact that phagedenic chancres or phagedenic ulceration of the rectum have not until recently, if at all, attracted in a prominent manner the attention of the profession. I don't now recollect what Mr. Allingham says about the subject; but Desprès, of Paris, was probably one of the first to point out, in a conspicuous manner, the relation between phagedenic ulceration of the rectum and stricture of this intestine. In my first article on "Stricture of the Rectum," in the *American Journal of the Medical Sciences*, of January, 1871, I alluded rather fully to the very important publication of Desprès, in the *Archives Générales de Medicine*, of March, 1868, on "Phagedenic Chancres of the Rectum.". My own article on "Rectal Stricture" was subsequently translated by, I believe, an Interne of the Paris Hospitals, into the *Archives.* At the time of the publication of this article, I had but a very imperfect practical acquaintance with phagedenic ulcerations of the rectum; indeed, Desprès' article alone afforded a positive confirmation of the alleged manner of the formation of stricture in most cases—namely, the appearance of soft chancres about the vulva and anus, and the extension of the sores into the rectum, and the resulting cicatricial tissue which causes a contraction of the gut, which varies with the more or less complete formation of a fibrous stricture of the rectum.

This phagedenic ulceration, however, occurs most frequently in extremely debilitated conditions of the system of syphilitic patients with ulceration of the rectum. Cicatrization of the ulcerated surface causes the stricture, and the stricture is not by any means a constitutional manifestation of syphilis—so there is an impropriety in the use of the term syphilitic stricture.

Although chancroidal ulceration of the rectum is an extremely frequent source of stricture, it must not be inferred that, with the exception of cancer, stricture of the rectum is always referable to syphilis as the original cause. In an article which I published in the *American Journal of the Medical Sciences* last July, the cause of stricture in one of my cases is clearly referred to the use of a pessary for a year without removal.

Unfortunately, the case of phagedenic ulceration was not seen again. Like many patients treated at the out-door departments of hospitals, and at dispensaries, this patient, from some cause, did not return as was expected.

### *CANCER OF THE VAGINA—THE UTERUS ATROPHIED, BUT OTHERWISE NORMAL.*

The following case of cancer of the vaginal walls was observed at my clinic at the Northwestern Dispensary of New York, December 9, 1871, and the notes of the case recorded by Dr. A. Landeta, in my case-book: Mrs. Anna Hawkins, aged fifty; one child eighteen years old; no miscarriages; menstruated at twelve years of age. Mrs. H. has a slightly purulent and sanguineous discharge from the vagina. She ceased to menstruate twelve years ago. The body and neck of the uterus are atrophied. To the vaginal touch there are nodulated indurations on the posterior surface of the vagina above the vaginal sphincter. To the rectal touch the rectum is healthy and empty. The uterine sound having been introduced into the uterus, it measured one and three-quarters to two inches. A speculum examination revealed the presence of superficial ulceration of the nodulated masses. The diagnosis was unmistakably malignant disease of the vaginal walls. I regret now, however, that a microscopic inspection of the secretions from the ulcerated surfaces was not made, in order to remove all doubt about the malignancy of this affection. The patient returned a few times, and was not again seen. Carbolized vaginal injections were prescribed.

The clinical features of this case were those of carcinoma, and my views were shared by Dr. Landeta. However, the unusual occurrence of cancer of the vaginal walls without the disease primarily affecting the uterus, is sufficient to require a very critical examination of the value of clinical symptoms of cancer under such circumstances.

The above reflections have been somewhat hastily thrown together, and, if not found to be unworthy of insertion in your valuable and popular journal, I shall be encouraged to again write to you in the same informal way.

Yours truly, WM. R. WHITEHEAD, M.D.,
*Late of New York.*

